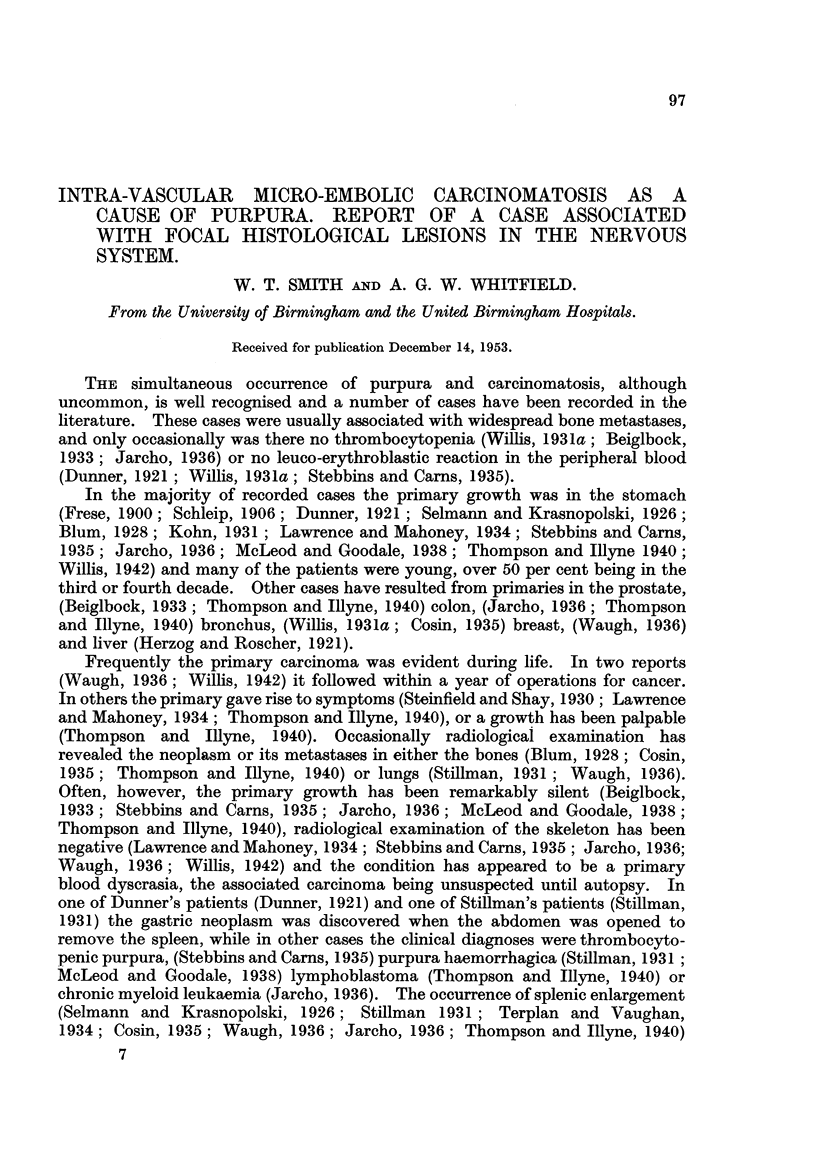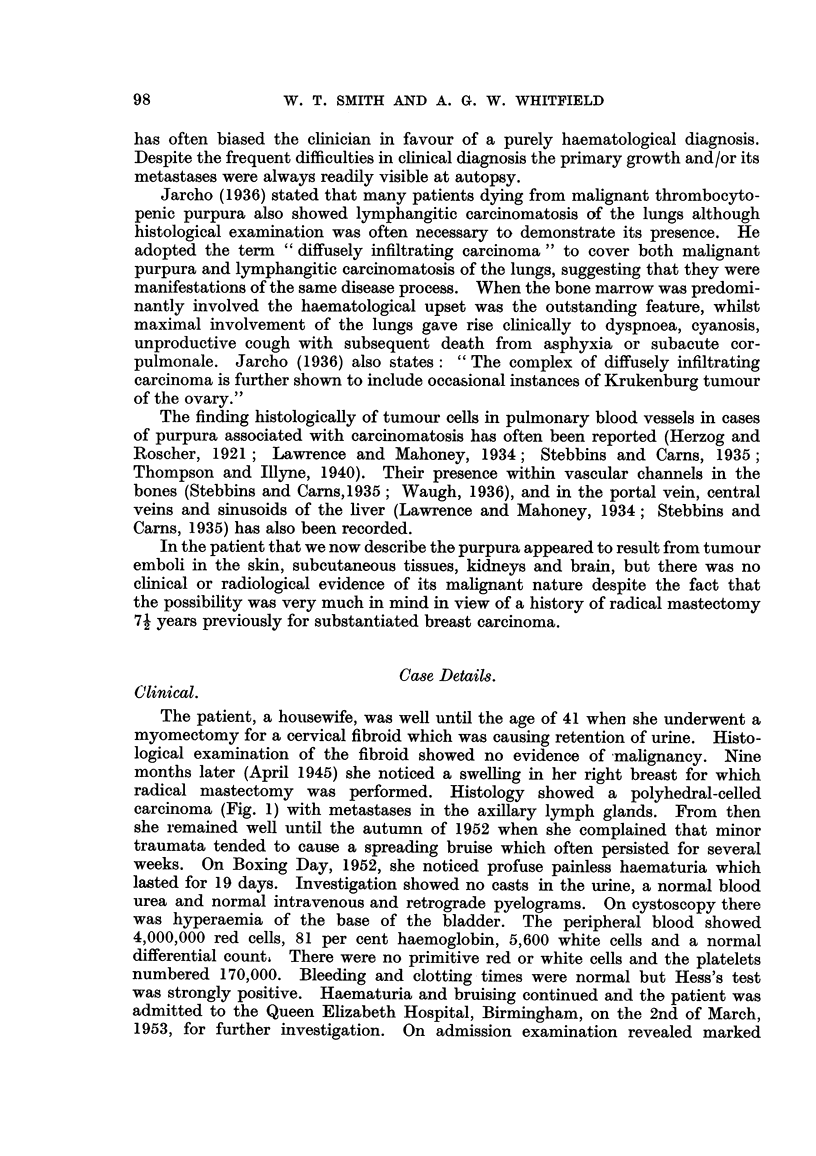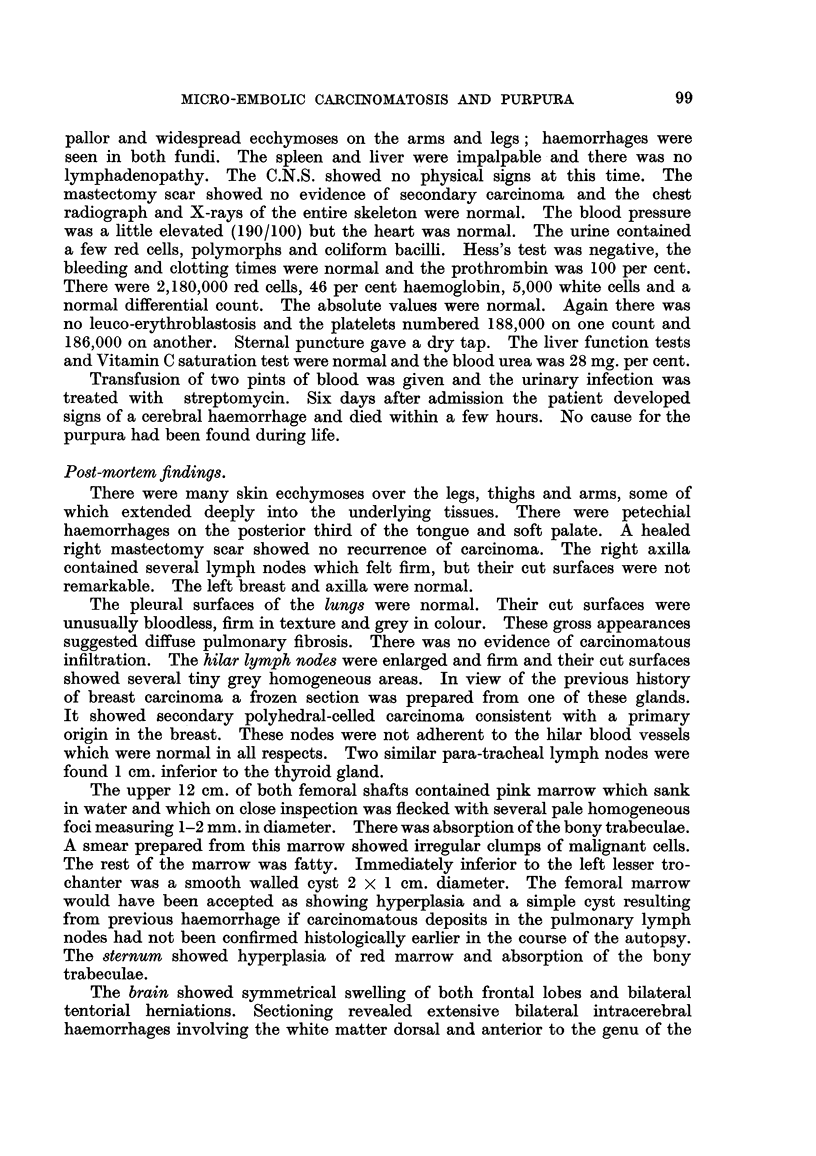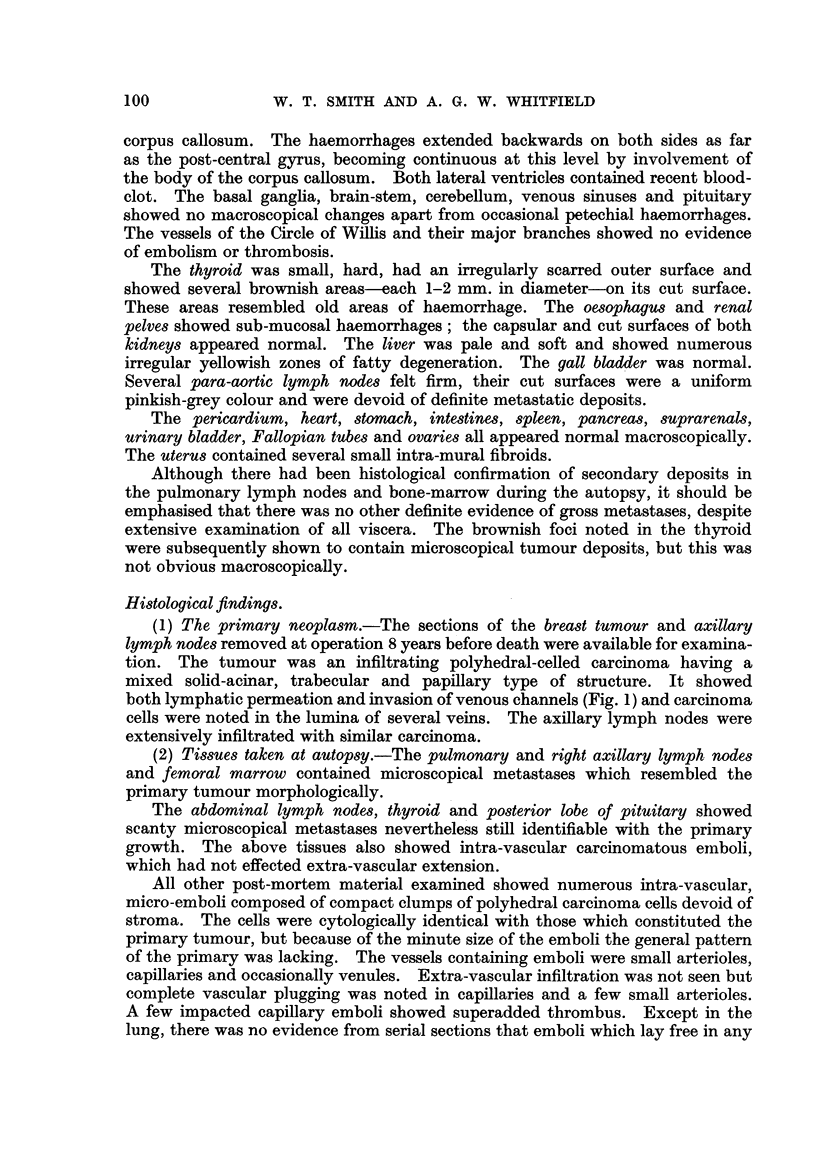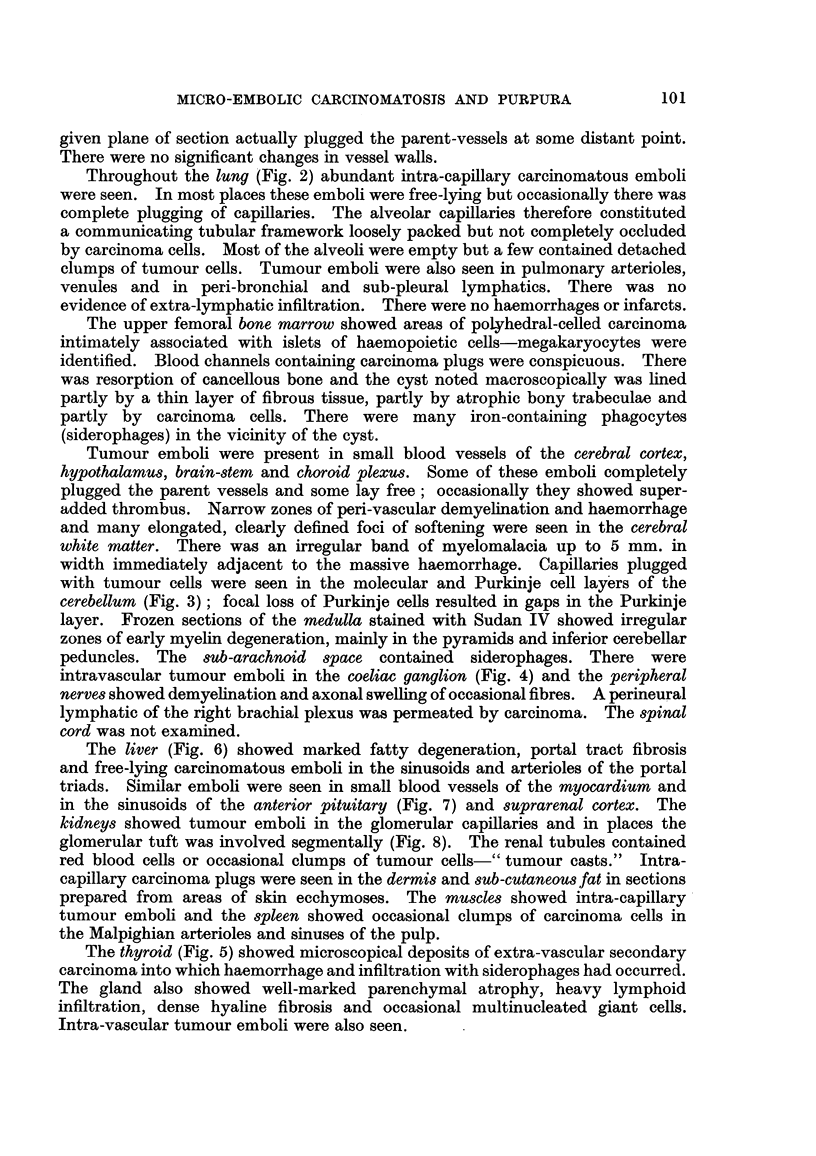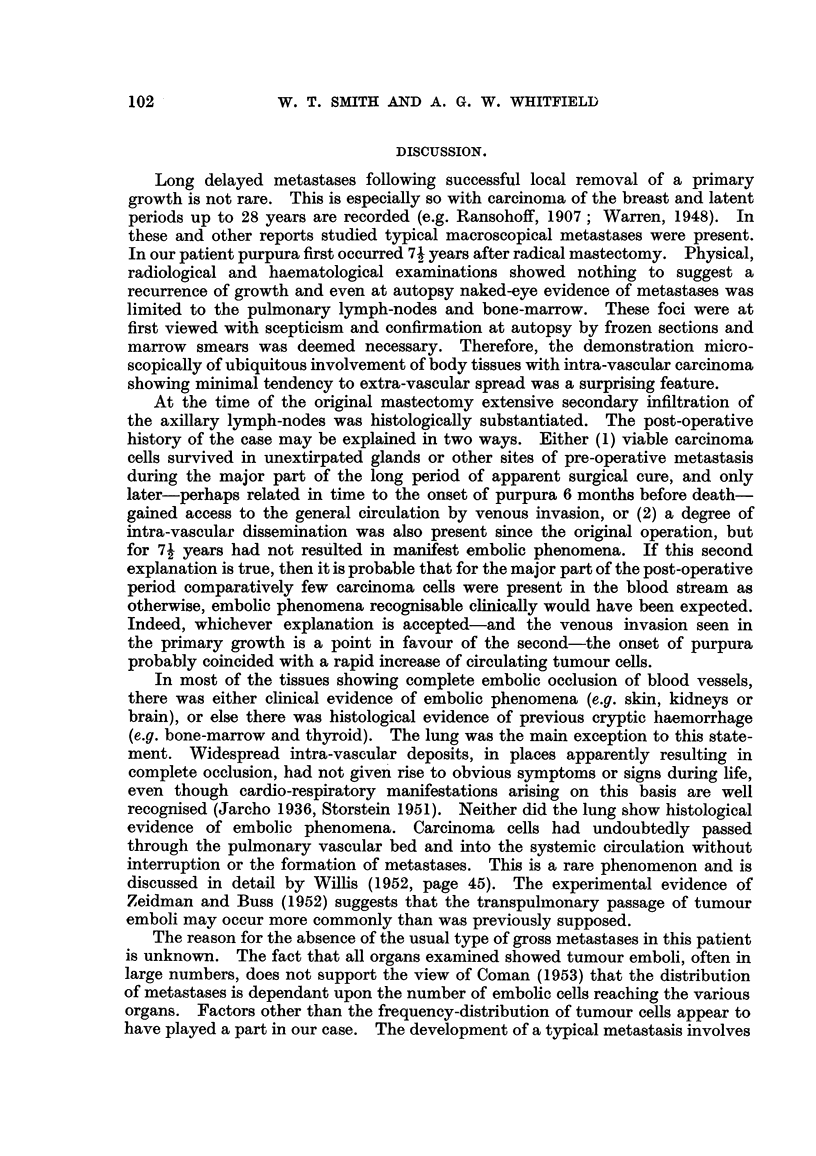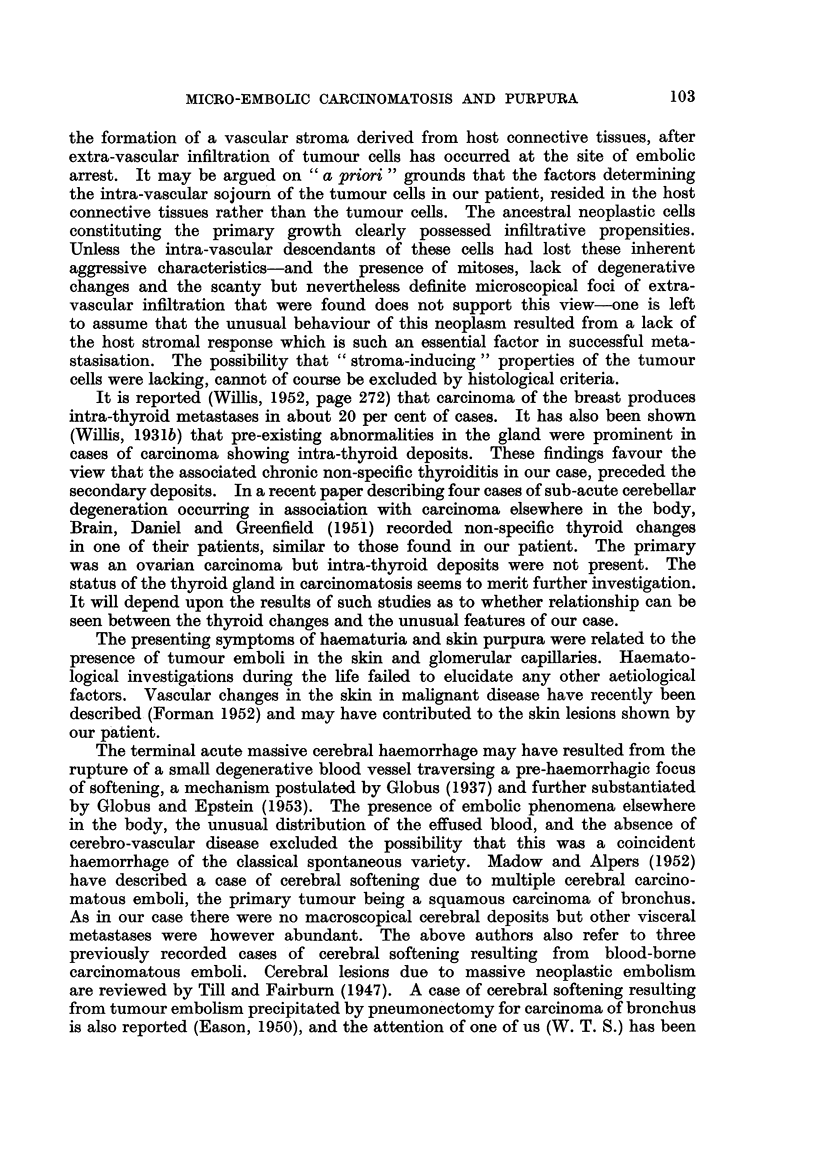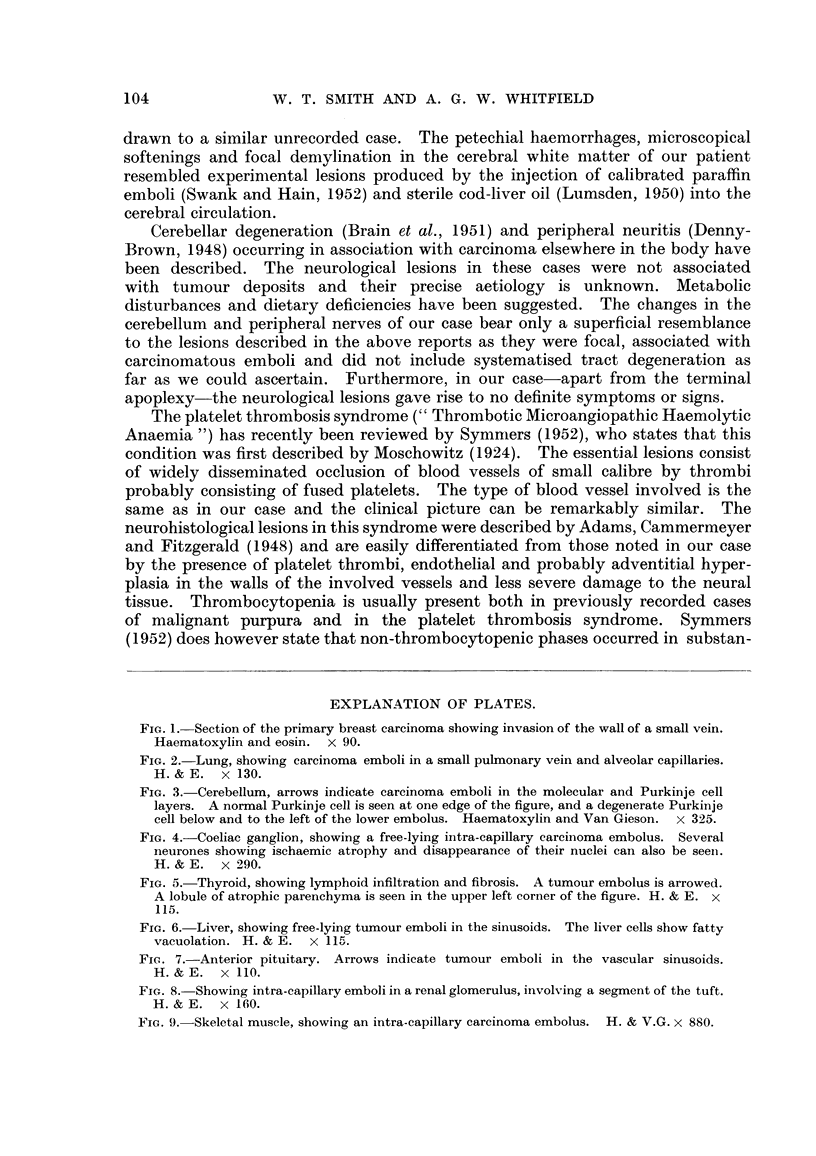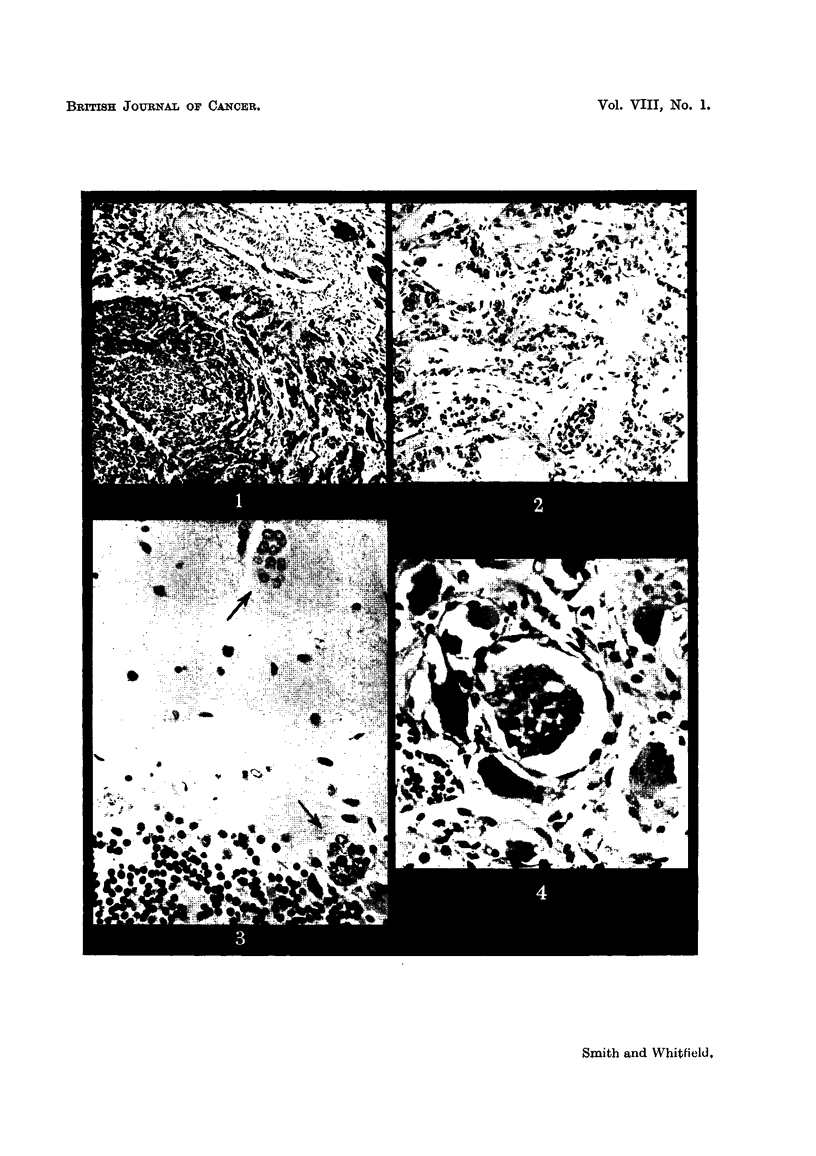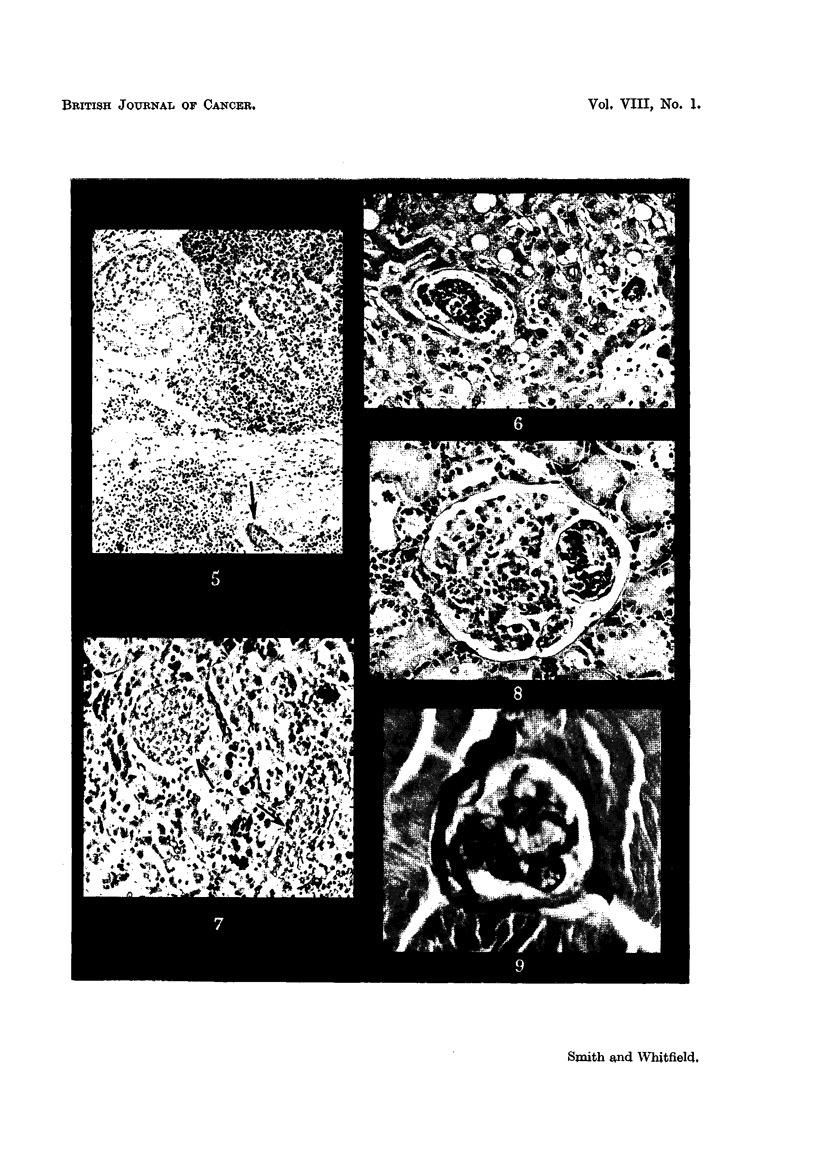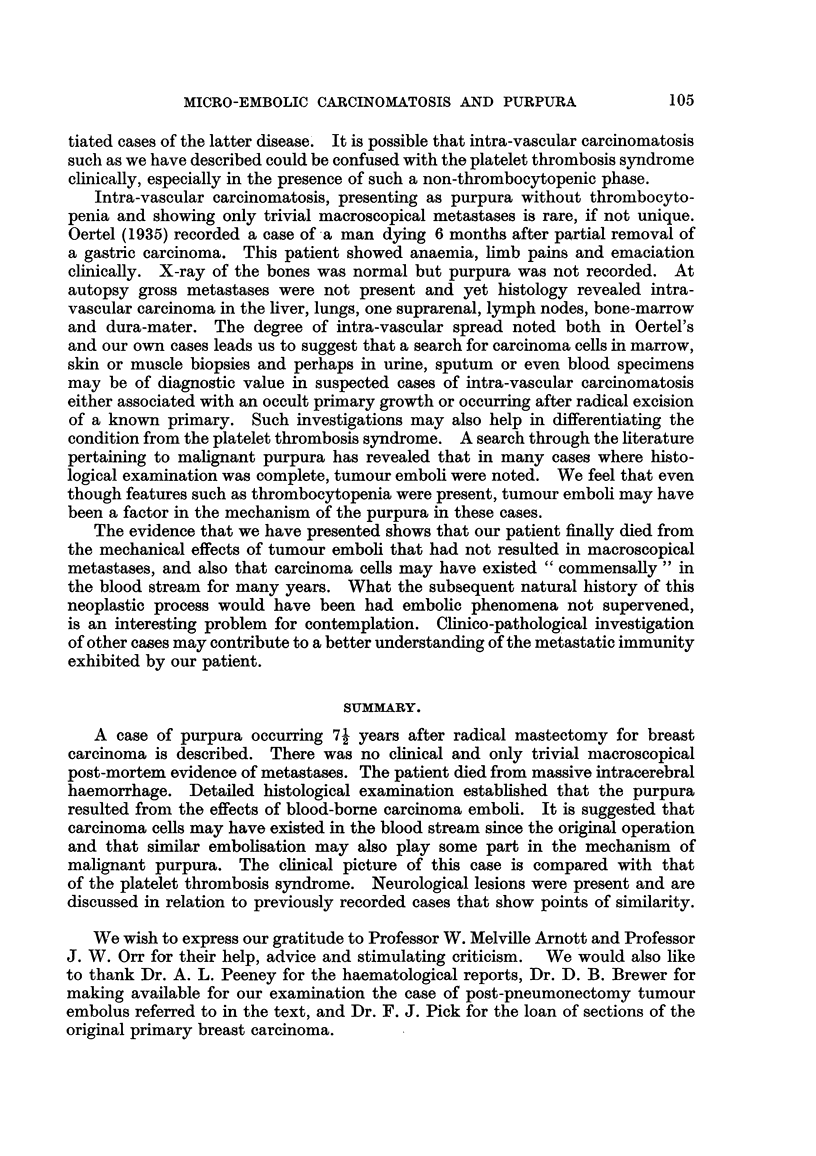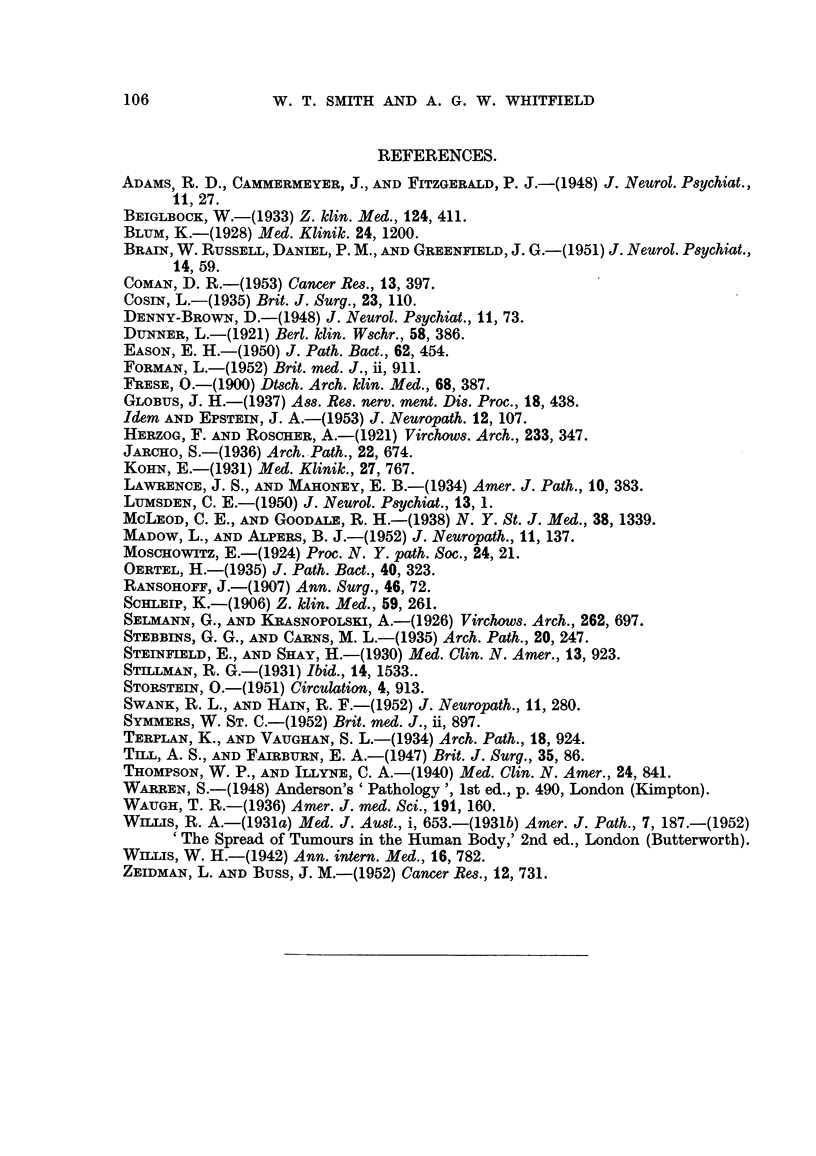# Intra-vascular Micro-embolic Carcinomatosis as a Cause of Purpura. Report of a Case Associated with Focal Histological Lesions in the Nervous System

**DOI:** 10.1038/bjc.1954.7

**Published:** 1954-03

**Authors:** W. T. Smith, A. G. W. Whitfield

## Abstract

**Images:**


### INTRA-VASCULAR MICRO-EMBOLIC CARCINOMATOSIS AS A CAUSE OF PURPURA. REPORT OF A CASE ASSOCIATED WITH FOCAL HISTOLOGICAL LESIONS IN THE NERVOUS SYSTEM.

W. T. SMITH AND A. G. W. WHITFIELD.

*From the University of Birmingham and the United Birmingham Hospitals.*

Received for publication December 14, 1953.

THE simultaneous occurrence of purpura and carcinomatosis, although uncommon, is well recognised and a number of cases have been recorded in the literature. These cases were usually associated with widespread bone metastases, and only occasionally was there no thrombocytopenia (Willis, 1931*a* ; Beiglbock, 1933 ; Jarcho, 1936) or no leuco-erythroblastic reaction in the peripheral blood (Dunner, 1921 ; Willis, 1931*a* ; Stebbins and Carns, 1935).

In the majority of recorded cases the primary growth was in the stomach (Frese, 1900 ; Schleip, 1906 ; Dunner, 1921 ; Selmann and Krasnopolski, 1926 ; Blum, 1928 ; Kohn, 1931 ; Lawrence and Mahoney, 1934 ; Stebbins and Carns, 1935 ; Jarcho, 1936 ; McLeod and Goodale, 1938 ; Thompson and Illyne 1940 ; Willis, 1942) and many of the patients were young, over 50 per cent being in the third or fourth decade. Other cases have resulted from primaries in the prostate, (Beiglbock, 1933 ; Thompson and Illyne, 1940) colon, (Jarcho, 1936 ; Thompson and Illyne, 1940) bronchus, (Willis, 1931*a* ; Cosin, 1935) breast, (Waugh, 1936) and liver (Herzog and Roscher, 1921).

Frequently the primary carcinoma was evident during life. In two reports (Waugh, 1936 ; Willis, 1942) it followed within a year of operations for cancer. In others the primary gave rise to symptoms (Steinfield and Shay, 1930 ; Lawrence and Mahoney, 1934 ; Thompson and Illyne, 1940), or a growth has been palpable (Thompson and Illyne, 1940). Occasionally radiological examination has revealed the neoplasm or its metastases in either the bones (Blum, 1928 ; Cosin, 1935 ; Thompson and Illyne, 1940) or lungs (Stillman, 1931 ; Waugh, 1936). Often, however, the primary growth has been remarkably silent (Beiglbock, 1933 ; Stebbins and Carns, 1935 ; Jarcho, 1936 ; McLeod and Goodale, 1938 ; Thompson and Illyne, 1940), radiological examination of the skeleton has been negative (Lawrence and Mahoney, 1934 ; Stebbins and Carns, 1935 ; Jarcho, 1936; Waugh, 1936 ; Willis, 1942) and the condition has appeared to be a primary blood dyscrasia, the associated carcinoma being unsuspected until autopsy. In one of Dunner's patients (Dunner, 1921) and one of Stillman's patients (Stillman, 1931) the gastric neoplasm was discovered when the abdomen was opened to remove the spleen, while in other cases the clinical diagnoses were thrombocytopenic purpura, (Stebbins and Carns, 1935) purpura haemorrhagica (Stillman, 1931 ; McLeod and Goodale, 1938) lymphoblastoma (Thompson and Illyne, 1940) or chronic myeloid leukaemia (Jarcho, 1936). The occurrence of splenic enlargement (Selmann and Krasnopolski, 1926 ; Stillman 1931 ; Terplan and Vaughan, 1934 ; Cosin, 1935 ; Waugh, 1936 ; Jarcho, 1936 ; Thompson and Illyne, 1940)

has often biased the clinician in favour of a purely haematological diagnosis. Despite the frequent difficulties in clinical diagnosis the primary growth and/or its metastases were always readily visible at autopsy.

Jarcho (1936) stated that many patients dying from malignant thrombocytopenic purpura also showed lymphangitic carcinomatosis of the lungs although histological examination was often necessary to demonstrate its presence. He adopted the term " diffusely infiltrating carcinoma " to cover both malignant purpura and lymphangitic carcinomatosis of the lungs, suggesting that they were manifestations of the same disease process. When the bone marrow was predominantly involved the haematological upset was the outstanding feature, whilst maximal involvement of the lungs gave rise clinically to dyspnoea, cyanosis, unproductive cough with subsequent death from asphyxia or subacute cor-pulmonale. Jarcho (1936) also states : " The complex of diffusely infiltrating carcinoma is further shown to include occasional instances of Krukenburg tumour of the ovary."

The finding histologically of tumour cells in pulmonary blood vessels in cases of purpura associated with carcinomatosis has often been reported (Herzog and Roscher, 1921 ; Lawrence and Mahoney, 1934; Stebbins and Carns, 1935; Thompson and Illyne, 1940). Their presence within vascular channels in the bones (Stebbins and Carns,1935 ; Waugh, 1936), and in the portal vein, central veins and sinusoids of the liver (Lawrence and Mahoney, 1934; Stebbins and Carns, 1935) has also been recorded.

In the patient that we now describe the purpura appeared to result from tumour emboli in the skin, subcutaneous tissues, kidneys and brain, but there was no clinical or radiological evidence of its malignant nature despite the fact that the possibility was very much in mind in view of a history of radical mastectomy $7\frac{1}{2}$ years previously for substantiated breast carcinoma.

### *Case Details.*

### *Clinical.*

The patient, a housewife, was well until the age of 41 when she underwent a myomectomy for a cervical fibroid which was causing retention of urine. Histological examination of the fibroid showed no evidence of malignancy. Nine months later (April 1945) she noticed a swelling in her right breast for which radical mastectomy was performed. Histology showed a polyhedral-celled carcinoma (Fig. 1) with metastases in the axillary lymph glands. From then she remained well until the autumn of 1952 when she complained that minor traumata tended to cause a spreading bruise which often persisted for several weeks. On Boxing Day, 1952, she noticed profuse painless haematuria which lasted for 19 days. Investigation showed no casts in the urine, a normal blood urea and normal intravenous and retrograde pyelograms. On cystoscopy there was hyperaemia of the base of the bladder. The peripheral blood showed 4,000,000 red cells, 81 per cent haemoglobin, 5,600 white cells and a normal differential count. There were no primitive red or white cells and the platelets numbered 170,000. Bleeding and clotting times were normal but Hess's test was strongly positive. Haematuria and bruising continued and the patient was admitted to the Queen Elizabeth Hospital, Birmingham, on the 2nd of March, 1953, for further investigation. On admission examination revealed marked pallor and widespread ecchymoses on the arms and legs; haemorrhages were seen in both fundi. The spleen and liver were impalpable and there was no lymphadenopathy. The C.N.S. showed no physical signs at this time. The mastectomy scar showed no evidence of secondary carcinoma and the chest radiograph and X-rays of the entire skeleton were normal. The blood pressure was a little elevated (190/100) but the heart was normal. The urine contained a few red cells, polymorphs and coliform bacilli. Hess's test was negative, the bleeding and clotting times were normal and the prothrombin was 100 per cent. There were 2,180,000 red cells, 46 per cent haemoglobin, 5,000 white cells and a normal differential count. The absolute values were normal. Again there was no leuco-erythroblastosis and the platelets numbered 188,000 on one count and 186,000 on another. Sternal puncture gave a dry tap. The liver function tests and Vitamin C saturation test were normal and the blood urea was 28 mg. per cent.

Transfusion of two pints of blood was given and the urinary infection was treated with streptomycin. Six days after admission the patient developed signs of a cerebral haemorrhage and died within a few hours. No cause for the purpura had been found during life.

*Post-mortem findings.*

There were many skin ecchymoses over the legs, thighs and arms, some of which extended deeply into the underlying tissues. There were petechial haemorrhages on the posterior third of the tongue and soft palate. A healed right mastectomy scar showed no recurrence of carcinoma. The right axilla contained several lymph nodes which felt firm, but their cut surfaces were not remarkable. The left breast and axilla were normal.

The pleural surfaces of the *lungs* were normal. Their cut surfaces were unusually bloodless, firm in texture and grey in colour. These gross appearances suggested diffuse pulmonary fibrosis. There was no evidence of carcinomatous infiltration. The *hilar lymph nodes* were enlarged and firm and their cut surfaces showed several tiny grey homogeneous areas. In view of the previous history of breast carcinoma a frozen section was prepared from one of these glands. It showed secondary polyhedral-celled carcinoma consistent with a primary origin in the breast. These nodes were not adherent to the hilar blood vessels which were normal in all respects. Two similar para-tracheal lymph nodes were found 1 cm. inferior to the thyroid gland.

The upper 12 cm. of both femoral shafts contained pink marrow which sank in water and which on close inspection was flecked with several pale homogeneous foci measuring 1–2 mm. in diameter. There was absorption of the bony trabeculae. A smear prepared from this marrow showed irregular clumps of malignant cells. The rest of the marrow was fatty. Immediately inferior to the left lesser trochanter was a smooth walled cyst 2 × 1 cm. diameter. The femoral marrow would have been accepted as showing hyperplasia and a simple cyst resulting from previous haemorrhage if carcinomatous deposits in the pulmonary lymph nodes had not been confirmed histologically earlier in the course of the autopsy. The *sternum* showed hyperplasia of red marrow and absorption of the bony trabeculae.

The *brain* showed symmetrical swelling of both frontal lobes and bilateral tentorial herniations. Sectioning revealed extensive bilateral intracerebral haemorrhages involving the white matter dorsal and anterior to the genu of the corpus callosum. The haemorrhages extended backwards on both sides as far as the post-central gyrus, becoming continuous at this level by involvement of the body of the corpus callosum. Both lateral ventricles contained recent blood-clot. The basal ganglia, brain-stem, cerebellum, venous sinuses and pituitary showed no macroscopical changes apart from occasional petechial haemorrhages. The vessels of the Circle of Willis and their major branches showed no evidence of embolism or thrombosis.

The *thyroid* was small, hard, had an irregularly scarred outer surface and showed several brownish areas—each 1–2 mm. in diameter—on its cut surface. These areas resembled old areas of haemorrhage. The *oesophagus* and *renal pelves* showed sub-mucosal haemorrhages ; the capsular and cut surfaces of both *kidneys* appeared normal. The *liver* was pale and soft and showed numerous irregular yellowish zones of fatty degeneration. The *gall bladder* was normal. Several *para-aortic lymph nodes* felt firm, their cut surfaces were a uniform pinkish-grey colour and were devoid of definite metastatic deposits.

The *pericardium, heart, stomach, intestines, spleen, pancreas, suprarenals, urinary bladder, Fallopian tubes* and *ovaries* all appeared normal macroscopically. The *uterus* contained several small intra-mural fibroids.

Although there had been histological confirmation of secondary deposits in the pulmonary lymph nodes and bone-marrow during the autopsy, it should be emphasised that there was no other definite evidence of gross metastases, despite extensive examination of all viscera. The brownish foci noted in the thyroid were subsequently shown to contain microscopical tumour deposits, but this was not obvious macroscopically.

*Histological findings.*

(1) *The primary neoplasm.*—The sections of the *breast tumour* and *axillary lymph nodes* removed at operation 8 years before death were available for examination. The tumour was an infiltrating polyhedral-celled carcinoma having a mixed solid-acinar, trabecular and papillary type of structure. It showed both lymphatic permeation and invasion of venous channels (Fig. 1) and carcinoma cells were noted in the lumina of several veins. The axillary lymph nodes were extensively infiltrated with similar carcinoma.

(2) *Tissues taken at autopsy.*—The *pulmonary* and *right axillary lymph nodes* and *femoral marrow* contained microscopical metastases which resembled the primary tumour morphologically.

The *abdominal lymph nodes, thyroid* and *posterior lobe of pituitary* showed scanty microscopical metastases nevertheless still identifiable with the primary growth. The above tissues also showed intra-vascular carcinomatous emboli, which had not effected extra-vascular extension.

All other post-mortem material examined showed numerous intra-vascular, micro-emboli composed of compact clumps of polyhedral carcinoma cells devoid of stroma. The cells were cytologically identical with those which constituted the primary tumour, but because of the minute size of the emboli the general pattern of the primary was lacking. The vessels containing emboli were small arterioles, capillaries and occasionally venules. Extra-vascular infiltration was not seen but complete vascular plugging was noted in capillaries and a few small arterioles. A few impacted capillary emboli showed superadded thrombus. Except in the lung, there was no evidence from serial sections that emboli which lay free in any given plane of section actually plugged the parent-vessels at some distant point. There were no significant changes in vessel walls.

Throughout the *lung* (Fig. 2) abundant intra-capillary carcinomatous emboli were seen. In most places these emboli were free-lying but occasionally there was complete plugging of capillaries. The alveolar capillaries therefore constituted a communicating tubular framework loosely packed but not completely occluded by carcinoma cells. Most of the alveoli were empty but a few contained detached clumps of tumour cells. Tumour emboli were also seen in pulmonary arterioles, venules and in peri-bronchial and sub-pleural lymphatics. There was no evidence of extra-lymphatic infiltration. There were no haemorrhages or infarcts.

The upper femoral *bone marrow* showed areas of polyhedral-celled carcinoma intimately associated with islets of haemopoietic cells—megakaryocytes were identified. Blood channels containing carcinoma plugs were conspicuous. There was resorption of cancellous bone and the cyst noted macroscopically was lined partly by a thin layer of fibrous tissue, partly by atrophic bony trabeculae and partly by carcinoma cells. There were many iron-containing phagocytes (siderophages) in the vicinity of the cyst.

Tumour emboli were present in small blood vessels of the *cerebral cortex*, *hypothalamus*, *brain-stem* and *choroid plexus*. Some of these emboli completely plugged the parent vessels and some lay free ; occasionally they showed super-added thrombus. Narrow zones of peri-vascular demyelination and haemorrhage and many elongated, clearly defined foci of softening were seen in the *cerebral white matter*. There was an irregular band of myelomalacia up to 5 mm. in width immediately adjacent to the massive haemorrhage. Capillaries plugged with tumour cells were seen in the molecular and Purkinje cell layers of the *cerebellum* (Fig. 3) ; focal loss of Purkinje cells resulted in gaps in the Purkinje layer. Frozen sections of the *medulla* stained with Sudan IV showed irregular zones of early myelin degeneration, mainly in the pyramids and inferior cerebellar peduncles. The *sub-arachnoid space* contained siderophages. There were intravascular tumour emboli in the *coeliac ganglion* (Fig. 4) and the *peripheral nerves* showed demyelination and axonal swelling of occasional fibres. A perineural lymphatic of the right brachial plexus was permeated by carcinoma. The *spinal cord* was not examined.

The *liver* (Fig. 6) showed marked fatty degeneration, portal tract fibrosis and free-lying carcinomatous emboli in the sinusoids and arterioles of the portal triads. Similar emboli were seen in small blood vessels of the *myocardium* and in the sinusoids of the *anterior pituitary* (Fig. 7) and *suprarenal cortex*. The *kidneys* showed tumour emboli in the glomerular capillaries and in places the glomerular tuft was involved segmentally (Fig. 8). The renal tubules contained red blood cells or occasional clumps of tumour cells—" tumour casts." Intra-capillary carcinoma plugs were seen in the *dermis* and *sub-cutaneous fat* in sections prepared from areas of skin ecchymoses. The *muscles* showed intra-capillary tumour emboli and the *spleen* showed occasional clumps of carcinoma cells in the Malpighian arterioles and sinuses of the pulp.

The *thyroid* (Fig. 5) showed microscopical deposits of extra-vascular secondary carcinoma into which haemorrhage and infiltration with siderophages had occurred. The gland also showed well-marked parenchymal atrophy, heavy lymphoid infiltration, dense hyaline fibrosis and occasional multinucleated giant cells. Intra-vascular tumour emboli were also seen.

DISCUSSION.

Long delayed metastases following successful local removal of a primary growth is not rare. This is especially so with carcinoma of the breast and latent periods up to 28 years are recorded (e.g. Ransohoff, 1907 ; Warren, 1948). In these and other reports studied typical macroscopical metastases were present. In our patient purpura first occurred 7½ years after radical mastectomy. Physical, radiological and haematological examinations showed nothing to suggest a recurrence of growth and even at autopsy naked-eye evidence of metastases was limited to the pulmonary lymph-nodes and bone-marrow. These foci were at first viewed with scepticism and confirmation at autopsy by frozen sections and marrow smears was deemed necessary. Therefore, the demonstration microscopically of ubiquitous involvement of body tissues with intra-vascular carcinoma showing minimal tendency to extra-vascular spread was a surprising feature.

At the time of the original mastectomy extensive secondary infiltration of the axillary lymph-nodes was histologically substantiated. The post-operative history of the case may be explained in two ways. Either (1) viable carcinoma cells survived in unextirpated glands or other sites of pre-operative metastasis during the major part of the long period of apparent surgical cure, and only later—perhaps related in time to the onset of purpura 6 months before death—gained access to the general circulation by venous invasion, or (2) a degree of intra-vascular dissemination was also present since the original operation, but for 7½ years had not resulted in manifest embolic phenomena. If this second explanation is true, then it is probable that for the major part of the post-operative period comparatively few carcinoma cells were present in the blood stream as otherwise, embolic phenomena recognisable clinically would have been expected. Indeed, whichever explanation is accepted—and the venous invasion seen in the primary growth is a point in favour of the second—the onset of purpura probably coincided with a rapid increase of circulating tumour cells.

In most of the tissues showing complete embolic occlusion of blood vessels, there was either clinical evidence of embolic phenomena (*e.g.* skin, kidneys or brain), or else there was histological evidence of previous cryptic haemorrhage (*e.g.* bone-marrow and thyroid). The lung was the main exception to this statement. Widespread intra-vascular deposits, in places apparently resulting in complete occlusion, had not given rise to obvious symptoms or signs during life, even though cardio-respiratory manifestations arising on this basis are well recognised (Jarcho 1936, Storstein 1951). Neither did the lung show histological evidence of embolic phenomena. Carcinoma cells had undoubtedly passed through the pulmonary vascular bed and into the systemic circulation without interruption or the formation of metastases. This is a rare phenomenon and is discussed in detail by Willis (1952, page 45). The experimental evidence of Zeidman and Buss (1952) suggests that the transpulmonary passage of tumour emboli may occur more commonly than was previously supposed.

The reason for the absence of the usual type of gross metastases in this patient is unknown. The fact that all organs examined showed tumour emboli, often in large numbers, does not support the view of Coman (1953) that the distribution of metastases is dependant upon the number of embolic cells reaching the various organs. Factors other than the frequency-distribution of tumour cells appear to have played a part in our case. The development of a typical metastasis involves the formation of a vascular stroma derived from host connective tissues, after extra-vascular infiltration of tumour cells has occurred at the site of embolic arrest. It may be argued on " *a priori* " grounds that the factors determining the intra-vascular sojourn of the tumour cells in our patient, resided in the host connective tissues rather than the tumour cells. The ancestral neoplastic cells constituting the primary growth clearly possessed infiltrative propensities. Unless the intra-vascular descendants of these cells had lost these inherent aggressive characteristics—and the presence of mitoses, lack of degenerative changes and the scanty but nevertheless definite microscopical foci of extra-vascular infiltration that were found does not support this view—one is left to assume that the unusual behaviour of this neoplasm resulted from a lack of the host stromal response which is such an essential factor in successful meta-stasisation. The possibility that " stroma-inducing " properties of the tumour cells were lacking, cannot of course be excluded by histological criteria.

It is reported (Willis, 1952, page 272) that carcinoma of the breast produces intra-thyroid metastases in about 20 per cent of cases. It has also been shown (Willis, 1931*b*) that pre-existing abnormalities in the gland were prominent in cases of carcinoma showing intra-thyroid deposits. These findings favour the view that the associated chronic non-specific thyroiditis in our case, preceded the secondary deposits. In a recent paper describing four cases of sub-acute cerebellar degeneration occurring in association with carcinoma elsewhere in the body, Brain, Daniel and Greenfield (1951) recorded non-specific thyroid changes in one of their patients, similar to those found in our patient. The primary was an ovarian carcinoma but intra-thyroid deposits were not present. The status of the thyroid gland in carcinomatosis seems to merit further investigation. It will depend upon the results of such studies as to whether relationship can be seen between the thyroid changes and the unusual features of our case.

The presenting symptoms of haematuria and skin purpura were related to the presence of tumour emboli in the skin and glomerular capillaries. Haemato-logical investigations during the life failed to elucidate any other aetiological factors. Vascular changes in the skin in malignant disease have recently been described (Forman 1952) and may have contributed to the skin lesions shown by our patient.

The terminal acute massive cerebral haemorrhage may have resulted from the rupture of a small degenerative blood vessel traversing a pre-haemorrhagic focus of softening, a mechanism postulated by Globus (1937) and further substantiated by Globus and Epstein (1953). The presence of embolic phenomena elsewhere in the body, the unusual distribution of the effused blood, and the absence of cerebro-vascular disease excluded the possibility that this was a coincident haemorrhage of the classical spontaneous variety. Madow and Alpers (1952) have described a case of cerebral softening due to multiple cerebral carcino-matous emboli, the primary tumour being a squamous carcinoma of bronchus. As in our case there were no macroscopical cerebral deposits but other visceral metastases were however abundant. The above authors also refer to three previously recorded cases of cerebral softening resulting from blood-borne carcinomatous emboli. Cerebral lesions due to massive neoplastic embolism are reviewed by Till and Fairburn (1947). A case of cerebral softening resulting from tumour embolism precipitated by pneumonectomy for carcinoma of bronchus is also reported (Eason, 1950), and the attention of one of us (W. T. S.) has been drawn to a similar unrecorded case. The petechial haemorrhages, microscopical softenings and focal demylination in the cerebral white matter of our patient resembled experimental lesions produced by the injection of calibrated paraffin emboli (Swank and Hain, 1952) and sterile cod-liver oil (Lumsden, 1950) into the cerebral circulation.

Cerebellar degeneration (Brain *et al.*, 1951) and peripheral neuritis (Denny-Brown, 1948) occurring in association with carcinoma elsewhere in the body have been described. The neurological lesions in these cases were not associated with tumour deposits and their precise aetiology is unknown. Metabolic disturbances and dietary deficiencies have been suggested. The changes in the cerebellum and peripheral nerves of our case bear only a superficial resemblance to the lesions described in the above reports as they were focal, associated with carcinomatous emboli and did not include systematised tract degeneration as far as we could ascertain. Furthermore, in our case—apart from the terminal apoplexy—the neurological lesions gave rise to no definite symptoms or signs.

The platelet thrombosis syndrome (" Thrombotic Microangiopathic Haemolytic Anaemia ") has recently been reviewed by Symmers (1952), who states that this condition was first described by Moschowitz (1924). The essential lesions consist of widely disseminated occlusion of blood vessels of small calibre by thrombi probably consisting of fused platelets. The type of blood vessel involved is the same as in our case and the clinical picture can be remarkably similar. The neurohistological lesions in this syndrome were described by Adams, Cammermeyer and Fitzgerald (1948) and are easily differentiated from those noted in our case by the presence of platelet thrombi, endothelial and probably adventitial hyperplasia in the walls of the involved vessels and less severe damage to the neural tissue. Thrombocytopenia is usually present both in previously recorded cases of malignant purpura and in the platelet thrombosis syndrome. Symmers (1952) does however state that non-thrombocytopenic phases occurred in substan-

---

### EXPLANATION OF PLATES.

Fig. 1.—Section of the primary breast carcinoma showing invasion of the wall of a small vein. Haematoxylin and eosin. × 90.

Fig. 2.—Lung, showing carcinoma emboli in a small pulmonary vein and alveolar capillaries. H. & E. × 130.

Fig. 3.—Cerebellum, arrows indicate carcinoma emboli in the molecular and Purkinje cell layers. A normal Purkinje cell is seen at one edge of the figure, and a degenerate Purkinje cell below and to the left of the lower embolus. Haematoxylin and Van Gieson. × 325.

Fig. 4.—Coeliac ganglion, showing a free-lying intra-capillary carcinoma embolus. Several neurones showing ischaemic atrophy and disappearance of their nuclei can also be seen. H. & E. × 290.

Fig. 5.—Thyroid, showing lymphoid infiltration and fibrosis. A tumour embolus is arrowed. A lobule of atrophic parenchyma is seen in the upper left corner of the figure. H. & E. × 115.

Fig. 6.—Liver, showing free-lying tumour emboli in the sinusoids. The liver cells show fatty vacuolation. H. & E. × 115.

Fig. 7.—Anterior pituitary. Arrows indicate tumour emboli in the vascular sinusoids. H. & E. × 110.

Fig. 8.—Showing intra-capillary emboli in a renal glomerulus, involving a segment of the tuft. H. & E. × 160.

Fig. 9.—Skeletal muscle, showing an intra-capillary carcinoma embolus. H. & V.G. × 880.

1

2

3

4

5

6

7

8

9

tiated cases of the latter disease. It is possible that intra-vascular carcinomatosis such as we have described could be confused with the platelet thrombosis syndrome clinically, especially in the presence of such a non-thrombocytopenic phase.

Intra-vascular carcinomatosis, presenting as purpura without thrombocytopenia and showing only trivial macroscopical metastases is rare, if not unique. Oertel (1935) recorded a case of a man dying 6 months after partial removal of a gastric carcinoma. This patient showed anaemia, limb pains and emaciation clinically. X-ray of the bones was normal but purpura was not recorded. At autopsy gross metastases were not present and yet histology revealed intra-vascular carcinoma in the liver, lungs, one suprarenal, lymph nodes, bone-marrow and dura-mater. The degree of intra-vascular spread noted both in Oertel's and our own cases leads us to suggest that a search for carcinoma cells in marrow, skin or muscle biopsies and perhaps in urine, sputum or even blood specimens may be of diagnostic value in suspected cases of intra-vascular carcinomatosis either associated with an occult primary growth or occurring after radical excision of a known primary. Such investigations may also help in differentiating the condition from the platelet thrombosis syndrome. A search through the literature pertaining to malignant purpura has revealed that in many cases where histological examination was complete, tumour emboli were noted. We feel that even though features such as thrombocytopenia were present, tumour emboli may have been a factor in the mechanism of the purpura in these cases.

The evidence that we have presented shows that our patient finally died from the mechanical effects of tumour emboli that had not resulted in macroscopical metastases, and also that carcinoma cells may have existed " commensally " in the blood stream for many years. What the subsequent natural history of this neoplastic process would have been had embolic phenomena not supervened, is an interesting problem for contemplation. Clinico-pathological investigation of other cases may contribute to a better understanding of the metastatic immunity exhibited by our patient.

### SUMMARY.

A case of purpura occurring 7½ years after radical mastectomy for breast carcinoma is described. There was no clinical and only trivial macroscopical post-mortem evidence of metastases. The patient died from massive intracerebral haemorrhage. Detailed histological examination established that the purpura resulted from the effects of blood-borne carcinoma emboli. It is suggested that carcinoma cells may have existed in the blood stream since the original operation and that similar embolisation may also play some part in the mechanism of malignant purpura. The clinical picture of this case is compared with that of the platelet thrombosis syndrome. Neurological lesions were present and are discussed in relation to previously recorded cases that show points of similarity.

We wish to express our gratitude to Professor W. Melville Arnott and Professor J. W. Orr for their help, advice and stimulating criticism. We would also like to thank Dr. A. L. Peeney for the haematological reports, Dr. D. B. Brewer for making available for our examination the case of post-pneumonectomy tumour embolus referred to in the text, and Dr. F. J. Pick for the loan of sections of the original primary breast carcinoma.

### REFERENCES.

ADAMS, R. D., CAMMERMEYER, J., AND FITZGERALD, P. J.—(1948) *J. Neurol. Psychiat.*, **11**, 27.
BEIGLBOCK, W.—(1933) *Z. klin. Med.*, **124**, 411.
BLUM, K.—(1928) *Med. Klinik.* **24**, 1200.
BRAIN, W. RUSSELL, DANIEL, P. M., AND GREENFIELD, J. G.—(1951) *J. Neurol. Psychiat.*, **14**, 59.
COMAN, D. R.—(1953) *Cancer Res.*, **13**, 397.
COSIN, L.—(1935) *Brit. J. Surg.*, **23**, 110.
DENNY-BROWN, D.—(1948) *J. Neurol. Psychiat.*, **11**, 73.
DUNNER, L.—(1921) *Berl. klin. Wschr.*, **58**, 386.
EASON, E. H.—(1950) *J. Path. Bact.*, **62**, 454.
FORMAN, L.—(1952) *Brit. med. J.*, ii, 911.
FRESE, O.—(1900) *Dtsch. Arch. klin. Med.*, **68**, 387.
GLOBUS, J. H.—(1937) *Ass. Res. nerv. ment. Dis. Proc.*, **18**, 438.
*Idem* AND EPSTEIN, J. A.—(1953) *J. Neuropath.* **12**, 107.
HERZOG, F. AND ROSCHER, A.—(1921) *Virchows. Arch.*, **233**, 347.
JARCHO, S.—(1936) *Arch. Path.*, **22**, 674.
KOHN, E.—(1931) *Med. Klinik.*, **27**, 767.
LAWRENCE, J. S., AND MAHONEY, E. B.—(1934) *Amer. J. Path.*, **10**, 383.
LUMSDEN, C. E.—(1950) *J. Neurol. Psychiat.*, **13**, 1.
McLEOD, C. E., AND GOODALE, R. H.—(1938) *N. Y. St. J. Med.*, **38**, 1339.
MADOW, L., AND ALPERS, B. J.—(1952) *J. Neuropath.*, **11**, 137.
MOSCHOWITZ, E.—(1924) *Proc. N. Y. path. Soc.*, **24**, 21.
OERTEL, H.—(1935) *J. Path. Bact.*, **40**, 323.
RANSOHOFF, J.—(1907) *Ann. Surg.*, **46**, 72.
SCHLEIP, K.—(1906) *Z. klin. Med.*, **59**, 261.
SELMANN, G., AND KRASNOPOLSKI, A.—(1926) *Virchows. Arch.*, **262**, 697.
STEBBINS, G. G., AND CARNS, M. L.—(1935) *Arch. Path.*, **20**, 247.
STEINFIELD, E., AND SHAY, H.—(1930) *Med. Clin. N. Amer.*, **13**, 923.
STILLMAN, R. G.—(1931) *Ibid.*, **14**, 1533..
STORSTEIN, O.—(1951) *Circulation*, **4**, 913.
SWANK, R. L., AND HAIN, R. F.—(1952) *J. Neuropath.*, **11**, 280.
SYMMERS, W. ST. C.—(1952) *Brit. med. J.*, ii, 897.
TERPLAN, K., AND VAUGHAN, S. L.—(1934) *Arch. Path.*, **18**, 924.
TILL, A. S., AND FAIRBURN, E. A.—(1947) *Brit. J. Surg.*, **35**, 86.
THOMPSON, W. P., AND ILLYNE, C. A.—(1940) *Med. Clin. N. Amer.*, **24**, 841.
WARREN, S.—(1948) Anderson's ' Pathology ', 1st ed., p. 490, London (Kimpton).
WAUGH, T. R.—(1936) *Amer. J. med. Sci.*, **191**, 160.
WILLIS, R. A.—(1931*a*) *Med. J. Aust.*, i, 653.—(1931*b*) *Amer. J. Path.*, **7**, 187.—(1952) ' The Spread of Tumours in the Human Body,' 2nd ed., London (Butterworth).
WILLIS, W. H.—(1942) *Ann. intern. Med.*, **16**, 782.
ZEIDMAN, L. AND BUSS, J. M.—(1952) *Cancer Res.*, **12**, 731.